# Interacting partners of *Brassica juncea* regulator of G-protein signaling protein suggest its role in cell wall metabolism and cellular signaling

**DOI:** 10.1042/BSR20220302

**Published:** 2022-07-14

**Authors:** Roshan Kumar, Naveen C. Bisht

**Affiliations:** Lab No. 106, National Institute of Plant Genome Research, Aruna Asaf Ali Marg, New Delhi 110067, India

**Keywords:** Heterotrimeric G-protein, Polyploid, Protein-protein interaction, Regulator of G-protein signaling, RGS, Yeast-two-hybrid

## Abstract

Heterotrimeric G-proteins interact with various upstream and downstream effectors to regulate various aspects of plant growth and development. G-protein effectors have been recently reported in *Arabidopsis thaliana*; however, less information is available from polyploid crop species having complex networks of G-protein components. Regulator of G-protein signaling (RGS) is a well-characterized GTPase accelerating protein, which plays an important role in the regulation of the G-protein cycle in plants. In the present study, four homologs encoding RGS proteins were isolated from the allotetraploid *Brassica juncea*, a globally important oilseed, vegetable, and condiment crop. The *B. juncea* RGS proteins were grouped into distinct BjuRGS1 and BjuRGS2 orthologous clades, and the expression of *BjuRGS1* homologs was predominantly higher than *BjuRGS2* homologs across the tested tissue types of *B. juncea*. Utilizing *B. juncea* Y2H library screening, a total of 30 nonredundant interacting proteins with the RGS-domain of the highly expressed BjuA.RGS1 was identified. Gene ontology analysis indicated that these effectors exerted various molecular, cellular, and physiological functions. Many of them were known to regulate cell wall metabolism (BjuEXP6, Bju-α-MAN, BjuPGU4, BjuRMS3) and phosphorylation-mediated cell signaling (BjuMEK4, BjuDGK3, and BjuKinase). Furthermore, transcript analysis indicated that the identified interacting proteins have a coexpression pattern with the *BjuRGS* homologs. These findings increase our knowledge about the novel targets of G-protein components from a globally cultivated *Brassica* crop and provide an important resource for developing a plant G-protein interactome network.

## Introduction

Heterotrimeric G-protein (hereafter G-protein) signaling plays a pivotal role in regulating various biological and cellular functions across phyla [1,2]. The core G-protein complex consists of Gα, Gβ, and Gγ subunits. In metazoans, ligand binding to G-protein coupled receptor (GPCR) facilitates the exchange of GTP for GDP on the Gα subunit, thereby dissociating the inactive core heterotrimer (Gαβy) complex into Gα-GTP and Gβy dimer [3,4]. Both these components interact separately with their effector proteins to regulate diverse downstream signaling pathways. Hydrolysis of Gα-GTP to Gα-GDP by the intrinsic GTP hydrolysis activity of Gα allows the reformation of the inactive heterotrimeric complex leading to the termination of the signal [5]. In addition, GTPase-accelerating activity of Regulator of G-protein Signaling (RGS) proteins further enhances the Gα-GTP hydrolysis, thereby desensitizing the G-protein-mediated signaling [6].

Although the core subunits of the G-protein complex remain the same across phyla, plants are known to possess unique regulation of G-protein signaling activation and deactivation. In the absence of typical GPCRs in plants, the activation of G-protein signaling relies primarily on the self-activating property of the Gα subunit. Additionally, in the plant kingdom, RGS protein acts as a crucial regulatory component that deactivates the G-protein signaling [2]. Plant RGS protein contains the N-terminal located ‘seven transmembrane (7-TM) structure’, which is absent from its animal counterparts, and the C-terminus cytoplasmic ‘RGS box’, which shows a high level of sequence similarity with archetypal RGS proteins [7]. The C-terminal located ‘RGS-box’ of plant RGS possesses the GAP activity [8–11].

Previous studies in *Arabidopsis* show the importance of sole RGS protein in regulating various plant growth and developmental processes like cell proliferation, germination, stomatal movement, sugar sensing, and response to phytohormone and various environmental cues [8,12–15]. The Arabidopsis *rgs1* mutant shows hyperactive responses like an increase in leaf and hypocotyl length. Ectopic expression of AtRGS1 confers smaller rosette size and shorter hypocotyl length [13]. Suppression of RGS protein leads to hyposensitive stress responses in *Arabidopsis* and mulberry [15,16]. In recent decades, tremendous progress has been made in elucidating the physiological responses and developmental phenotypes of plant G-protein components; however, the intricacies of the molecular cascade associated with these responses are yet to be discovered.

In a multicellular system, protein–protein interactions (PPIs) play a very crucial role in regulating various cellular processes. Yeast-two-hybrid (Y2H) has been widely used for the identification of the interacting partners of proteins associated with complex regulatory pathways [17–19]. Compared with metazoans, limited sets of effector molecules for G-protein components have been identified from plants, particularly from crop species, and their characterization is still in the infancy stage. In an earlier study using Y2H and proteomic-based screening approaches, various targets of core G-protein components have been reported from the model plant *Arabidopsis* [17,18,20]. Recently, Y2H screening of multiple Gβ subunits (paralogs) of *Brassica juncea* led to the identification of both subunit-specific and common interacting partners, that are known to control a wide range of cellular and biological processes [21].

*B. juncea* (AABB, 2*n*=36) is a natural interspecific hybrid between *Brassica rapa* (AA, 2*n*=20) and *Brassica nigra* (BB, 2*n*=16) [22,23]. It is grown worldwide as an important oilseed, vegetable, and condiment crop. Evolutionary events like whole-genome triplication (WGT), allopolyploidization, and genomic rearrangements in *Brassica* lineage have created multiple homeologs shaping tremendous morphological, developmental, and chemical plasticity across *Brassica* crop species [24,25]. Since G-protein signaling components control various aspects of plant growth and development [2], identification of the G-protein-effectors could be crucial to outlining the regulatory networks involved in multiple biological and cellular functions.

In the present study, we identified multiple homologs of RGS proteins from the allotetraploid *B. juncea.* Furthermore, a well-expressed *B. juncea* RGS isoform was selected as bait protein to screen the Y2H cDNA library constructed from mRNA isolated from reproductive tissues of *B. juncea.* A large number of RGS-interacting proteins were identified, and Gene Ontology (GO) analysis predicted their involvement in a wide range of biological and molecular functions. Furthermore, coexpression analysis of the RGS-interacting partners is also presented. These findings increase our knowledge of the RGS-interacting proteins and provide a resource for developing a plant G-protein interactome network.

## Materials and methods

### Plant material and growth conditions

In the present study, *B. juncea* L. (cv. Varuna) was grown under short-day conditions (10 h light/14 h dark) at 24°C with a photon flux density of approximately 300 μmol m^−2^ s^−1^ and 60–70% relative humidity. Tissue types representing different developmental stages like 5-day-old seedlings, root, stem, young leaves from 1-month-old plants, flower (unopen flower buds), and developing siliques (7 and 14 days-after-pollination (DAP)) were collected and stored at −80°C.

### Amplification, cloning, and phylogenetic analysis of full-length *RGS* coding DNA sequences from *B. juncea*

Gene-specific primers (Supplementary Table S1) were designed based on our previously reported *RGS* sequences from *Brassica* species [11], and the full-length RGS coding DNA sequences (CDS) were amplified from *B. juncea*. Following PCR amplification, the PCR products were cloned into a pENTR/D-TOPO vector (Invitrogen) and sequenced to determine the accuracy and identity of the clones. Phylogenetic analysis was carried out using deduced RGS sequences of *B. juncea* and those retrieved from the order Brassicales (https://phytozome.jgi.doe.gov/pz/portal.html) using the neighbor-joining method with 1000 bootstrap iterations in MEGAX-32 [26].

### Total RNA isolation, cDNA synthesis, and real-time qRT-PCR

Total RNA isolation was from different vegetative and reproductive developmental tissues (seedling, root, leaves, stem, flower, siliques) of *B. juncea*. The first-strand cDNA synthesis and qRT-PCR were conducted in the same manner as previously described [27]. The cDNA samples representing different stages of plant growth and development were diluted to 1:20 and qRT-PCR was performed using gene-specific primers (Supplementary Table S1).

### Construction of ‘BjuA.RGS1box + Ct’ bait vector

To construct a bait vector for the Y2H analysis, the CDS of BjuA.RGS1box along with C-terminal (BjuA.RGS1box+Ct domain) was amplified with primers containing the restriction sites of *NcoI* and *EcoRI* (Supplementary Table S1) and then cloned into bait vector ‘pGBKT7’ harboring GAL4 DNA-binding domain (BD). Subsequently, the bait vector pGBKT7-BjuA.RGS1box+Ct domain was introduced into yeast strain Y2H-Gold using PEG/LiAc-mediated yeast transformation and plated on a minimal medium lacking tryptophan (SD/−Trp). Furthermore, autoactivation of pGBKT7-BjuA.RGS1box+Ct domain bait construct was tested on SD/−Trp plates supplemented with X-α-Gal (40 mg ml^−1^) and Aureobasidin A (125 ng ml^−1^).

### Screening and identification of BjuA.RGS1-interacting proteins

The construction of *B. juncea* Y2H library used in the present study was previously reported [21], and was developed from the RNA isolated from reproductive stages (flowers and developing siliques) of *B. juncea*. For screening the interacting proteins of BjuA.RGS1, the bait strain cells containing the pGBKT7-BjuA.RGS1box+Ct domain were mated with *B. juncea* prey library cells in 2X YPDA liquid medium and incubated for 28 h at 30°C. The mated culture was plated onto SD/-Leu/-Trp (double drop-out (DDO)) plates containing X-α-Gal (40 mg ml^−1^) and Aureobasidin A (125 ng ml^−1^) and kept at 30°C for 5 days. Moreover, 100 µl of the mated culture was also plated on SD/-Leu and SD/-Lue/-Trp medium in different dilutions (1/10, 1/100, 1/1000, and 1/10000), and mating efficiency was calculated by the number of CFU/ml of diploids divided by CFU/ml of limiting factor (prey libraries) × 100 on SD/-Leu plate. Furthermore, colonies grown for 4–5 days (blue colonies) obtained on DDO/X/A were streaked onto the higher stringent medium SD/-Leu/-Trp/-Ade/-His (quadruple drop out (QDO)) containing X-α-Gal (40 mg ml^−1^) and Aureobasidin A (125 ng ml^−1^) (QDO/X/A).

Furthermore, yeast colony PCR was performed on colonies showing activation of all the reporter genes and PCR products were sequenced. To identify the interacting proteins, sequences were analyzed using BLAST analysis in BRAD (http://brassicadb.org/brad/blastPage.php) and NCBI (http://blast.ncbi.nlm.nih.gov/Blast.cgi) databases. Furthermore, functional annotation of the identified BjuA.RGS1-interacting proteins was obtained after GO analysis using PlantGSEA (Plant GeneSet Enrichment Analysis) with *A. thaliana* used as the background genome [28]. The GO terms enrichments were carried out using Fisher’s test and Benjamini–Hochberg (false-discovery rate cutoff of 0.05) correction applied for calculation of adjusted *P*-values. Thereafter, REVIGO was used to visualize the interactive graph of over-represented GO terms [29]

## Results and discussion

### Isolation and expression analysis of *B. juncea RGS* genes

**Figure 1:**
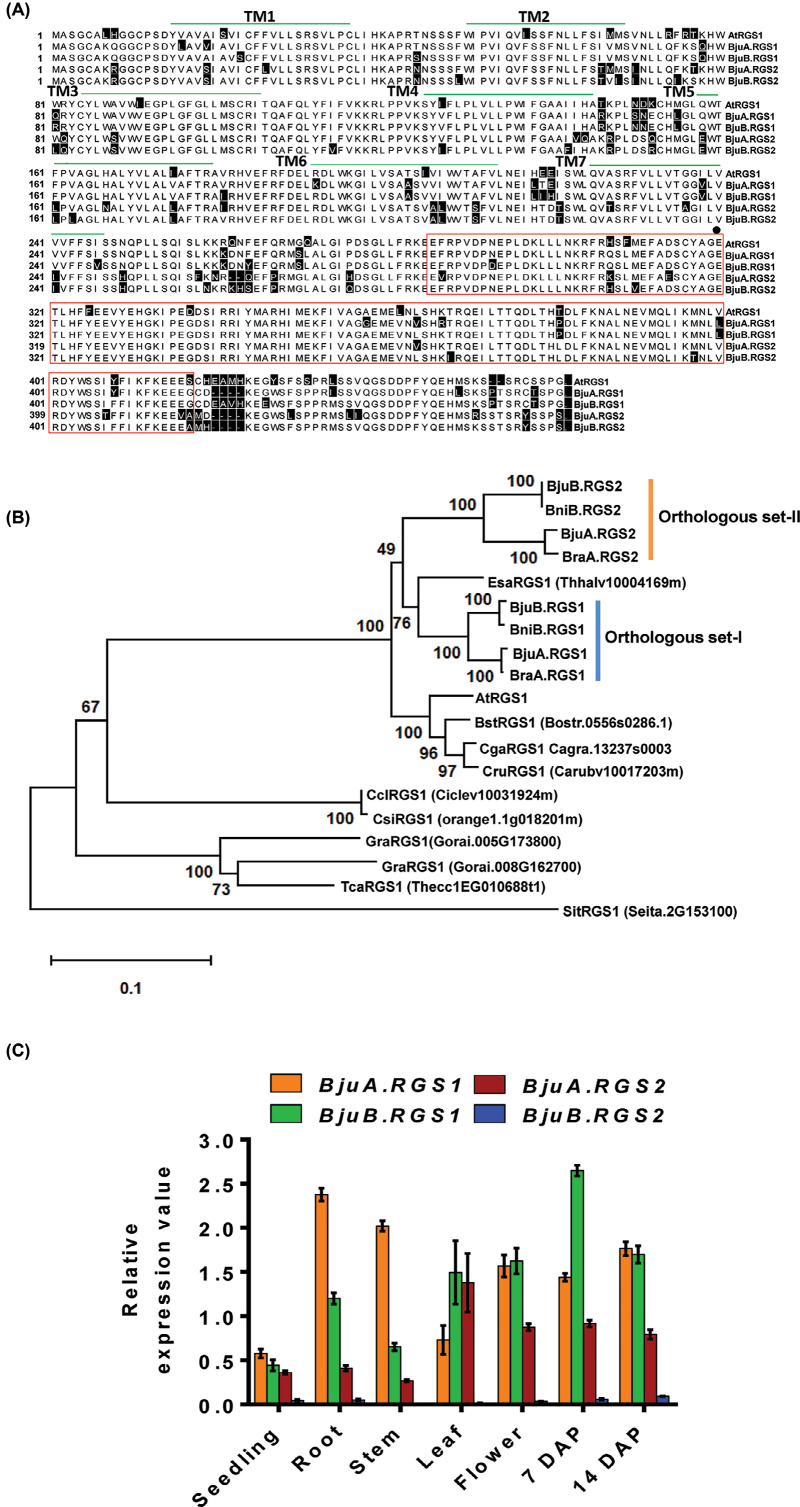
Sequence analysis and transcript expression of *B. juncea* RGS genes (**A**) Amino acid sequence alignment of the deduced BjuRGS proteins was performed using ClustalW in the software DNASTAR. Divergent amino acid residues are indicated by a black shadow. The predicted 7-TM domains are marked within the horizontal green lines and RGS-box is shown within the red box. The critical Glu (E) residue for GAP activity of RGS protein is indicated with a filled circle. (**B**) The phylogenetic analysis was inferred using the Neighbor-Joining method in MEGA-X-32. The tree was constructed using 19 RGS protein sequences and the evolutionary distances were computed using the Poisson correction method. Numbers above branches of the tree indicate the percentage of bootstrap values. (**C**) Transcript expression profile of *BjuRGS* genes at different developmental stages of *B. juncea*. The expression data were normalized against *B. juncea Actin* (set at 100). Data represent the mean ± SE of four independent measurements.

In the present study, four full-length *RGS* homologs (*BjuA.RGS1*, *BjuA.RGS2*, *BjuB.RGS1*, and *BjuB.RGS2*) like sequences were isolated from *B. juncea* and cloned into pENTR/D-TOPO entry vector. The *B. juncea RGS* homologs were classified and named based on the sequences obtained from its progenitor genomes, i.e. *B. rapa* and *B. nigra*. The *BjuA.RGS1* and *BjuA.RGS2* corresponds to A-genome-specific *BraA.RGS1* and *BjuA.RGS2*, respectively; whereas *BjuB.RGS1* and *BjuB.RGS2* correspond to *BniB.RGS1* and *BniB.RGS2*, respectively. Full-length coding *RGS* sequences isolated from *B. juncea* ranged from 1368 to 1386 bp, encoding proteins of 455–461 amino acids in length. Deduced RGS proteins of *B. juncea* shared 84.6–95.2% identity among themselves and 84–88.9% identity with the *A. thaliana* AtRGS1 (Supplementary Table S2). Amino acid sequence alignment showed that the *B. juncea* RGS proteins contain the N-terminal ‘7-TM domain’ and the C-terminal located ‘RGS domain’, and share high sequence conservation with orthologs from *B. rapa* and *B. nigra* (Figure 1A) [11]. Furthermore, a key residue (Glu320) responsible for the GAP activity of Arabidopsis RGS protein was also found to be highly conserved in BjuRGS proteins [9]. Phylogenetic analysis of RGS proteins belonging to core Brassicaceae revealed that the BjuRGS proteins grouped into two independent orthologous clades, one containing the RGS1 and other RGS2 proteins (Figure 1B).

To get the primary insight into the role of multiple *RGS* homologs in *B. juncea*, we further determined the expression level of *BjuRGS* genes across various stages of plant growth and development. The qRT-PCR analysis showed that all the members of *B. juncea RGS* genes were expressed although showing differential expression patterns (Figure 1C). The *BjuA.RGS1* and *BjuB.RGS1* were found to be highly expressed genes with profound expression observed in the root, flower, and siliques stages as also reported for the *B. rapa* ortholog, *BraA.RGS1* [11]. The *BjuB.RGS2* transcript had low abundance across all the examined tissue types. Structural and gene expression changes are an important characteristic of polyploidy, and duplicated genes, i.e. homeologs often tend to possess differential expression patterns [30,31]. *B. juncea* is an allopolyploid, formed by the fusion of two different genomes. In the present study, the differential expression pattern was observed for the BjuRGS homologs across different developmental stages. Interestingly, the expression of type-I (*BjuRGS1*) homologs that shared the close phylogeny with *Arabidopsis* counterpart (AtRGS1) was predominantly higher than *BjuRGS2* homologs (Figure 1B). Overall, the expression data suggest the possible functional dominance of type-I *RGS* homeologs in the allopolyploid *B. juncea*. The expression dominance of G-protein gene homeologs is quite a norm in polyploid plant species like soybean and *Brassica*, and this phenomenon is known to regulate the G-protein activities and biological functions [10,11,21,32].

### Identification of BjuA.RGS1-interacting proteins by Y2H screening

It is well established that only the C-terminal region (Ct) of RGS protein possesses the GAP [8,9]. Therefore, to carry out the Y2H screening, the Ct of the ubiquitously expressed group-I RGS protein, i.e. BjuA.RGS1 was selected for the screening. The BjuA.RGS1box including its Ct was cloned into a pGBKT7 bait vector. The yeast cells containing bait clone alone were unable to grow on the selection plate (DDO/X/A), indicating that BjuA.RGS1box+Ct domain does not display autoactivation property. Further mating efficiency was determined as 5.3%, which was within the range of 2–5%, as per the manufacturer’s protocol. A total of approximately 2000 blue colonies were obtained on the double drop-out selection medium (DDO/X/A) (Table 1). Furthermore, these colonies were patched on a more stringent quadruple drop out (QDO/X/A) minimal medium, which resulted in optimum growth of more than 1900 colonies.

**Table 1:** Summary of *B. juncea* Y2H cDNA library screened using BjuA.RGS1box+Ct domain as the bait

Measured parameters	Count
Library titer (cfu/ml)	7.5 × 10^7^
Mating efficiency (%)	5.3
No. of diploids obtained on DDO/X/A	∼2000
No. of diploids obtained on QDO/X/A	∼1900
No. of diploids screened using colony PCR	∼1000
No. of unique proteins	51
No. of genuine-interacting proteins (in-frame)	30
No. of false-positive/nonframe clones	21

**Figure 2:**
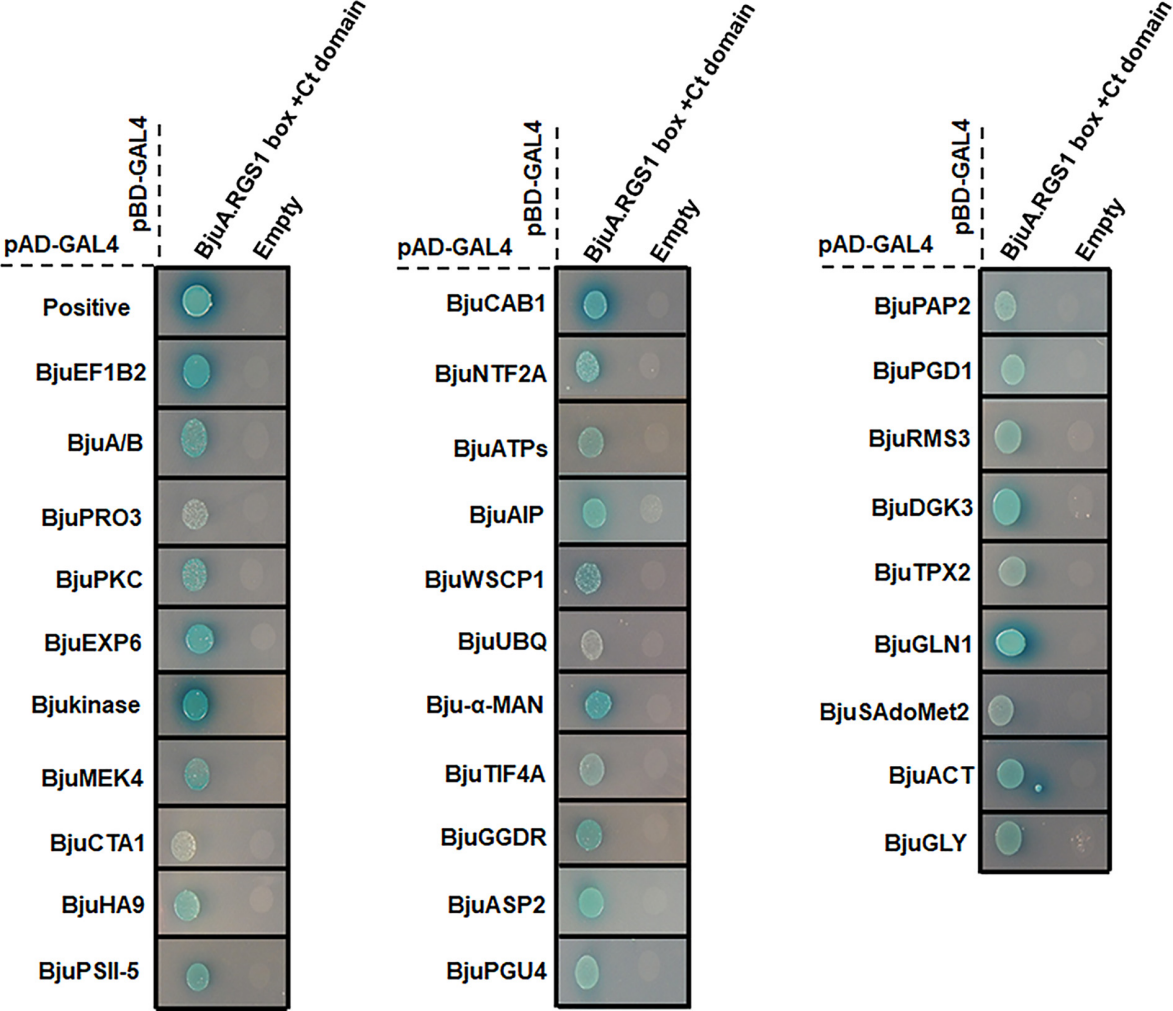
Y2H screening of *B. juncea* cDNA library using BjuA.RGS1box+Ct domain CDS of BjuA.RGS1 box with its Ct was cloned in the pGBKT7 bait vector to screen the cDNA library. The interaction was tested and verified by cell growth assay on a QDO medium with 40 mg ml^−1^ X-α-gal and 200 ng ml^−1^ Aureobasidin A. Cotransformation with pGBKT7-53 and pGADT7-T was used as a positive control, while cotransformation of pGBKT7-empty vector and pGADT7-prey plasmids was used as empty control.

Furthermore, colony PCR was performed using primers for the pGAL4-AD prey vector (Supplementary Table S1), and a total of 1000 blue clones were sequenced to reveal their identity. Sequence analyses using BLASTn revealed 51 nonredundant clones as the putative-interacting partners of BjuA.RGS1 protein. To rule out autoactivation and re-examine the interaction, rescued prey plasmids were individually cotransformed into yeast strain Y2H-Gold along with bait (pGBKT7-BjuA.RGS1box+Ct domain) or empty vector (pGBKT7). The one-to-one Y2H assay further confirmed 30 out of 51 prey plasmids to be genuine interactors of BjuA.RGS1box+Ct domain (Figure 2), while the remaining 21 prey plasmids either displayed autoactivation or were not in-frame (Table 1). Furthermore, to validate the positive and genuine interaction, a one-to-one Y2H assay was performed using 15 prey plasmids (representing various important biological functions) cotransformed with pGBKT7-BjuA.RGS1 box+Ct domain or pGBKT7 empty vector into Y2HGold strain (Supplementary Figure S1). The selection was carried out on growing diploid yeast cells on a QDO medium containing different concentrations of 3-Amino-1.2.4-triazole (3-AT). The 3-AT is a competitive inhibitor of *HIS3* gene product used as a reporter in Y2H and is typically added to select the strong interactions and eliminate the false-positive results. The tested combination shows growth on the QDO+3-AT medium, therefore, ruling out the possibility of false-negative interaction. Overall, several of the identified BjuA.RGS1box+Ct domain-interacting proteins showed a differential level of interaction strength, as noted by growth and blue color intensity on different selection mediums (Figure 1 and Supplementary Figure S1).

### Functional annotation and transcript expression analysis of BjuA.RGS1-interacting proteins

As the information about the genomic resources for *Brassica* species is limited, functional annotation of the interacting proteins identified for BjuA.RGS1box+Ct domain was performed using the GO tool of the TAIR database. The interacting proteins identified in the present study are novel targets of plant RGS, showing involvement in various biological and cellular functions (Table 2). Furthermore, functional enrichment analysis was also carried out using the PlantGSEA using *A. thaliana* whole genome annotation as the background. TAIR gene IDs were submitted to PlantGSEA and GO terms with their associated significance values (*P*-values) were obtained (Supplementary Table S3). REVIGO visualization of over-represented GO terms grouped the interacting proteins based on their biological process, molecular function, and cellular component (Supplementary Figure S2). In the biological process, the most enriched GO terms are associated with BraA.RGS1-interacting proteins were metabolism, ion transport, and cytoskeleton organization. In terms of molecular function, GO terms were associated with nucleotide and amino acid binding, kinase activity, oxidoreductase activity, α-mannosidase activity, and transporter activity. The cellular compartment category revealed the distribution of proteins into various cellular and subcellular categories, namely membrane, cytosol, cell wall, cell junction, and plastid.

**Table 2:** List of interacting partners identified by Y2H analysis using BjuA.RGS1box+Ct domain as bait

S. No	Gene symbols	Gene locus (BRAD)	Protein name	UniProtKB name	Homolog function in *Arabidopsis thaliana*
1	*BjuEF1B2*	BjuA009282	Elongation factor 1-beta 2	Q9SCX3	Guanyl-nucleotide exchange factor activity
2	*BjuA/B*	BjuB013044	A/B barrel domain-containing protein	Q9LUV2	Defense against fungal pathogens
3	*BjuPRO3*	BjuB016067	Profilin 3	Q38904	Cytoskeleton organization
4	*BjuPKC*	BjuB031223	Polyketide cyclase	F4J2V1	Lipid binding
5	*BjuEXP6*	BjuA026347	Expansin A6	Q38865	Cell wall loosening
6	*Bjukinase*	NA	Serine/threonine-protein kinase	Q9MAB4	Protein phosphorylation activity
7	*BjuMEK4*	BjuA002904	Mitogen-activated protein kinase kinase 4	O80397	Protein phosphorylation activity
8	*BjuCT1A*	BjuB011087	Curvature thylakoid 1a	B3H429	Not known
9	*BjuHA9*	BjuB022127	H[+]-ATPase 9	Q1PFB6	Biosynthetic process
10	*BjuPSII-5*	BjuB021812	Photosystem II 5 kDa protein	Q0WWI7	Biosynthetic process
11	*BjuCAB1*	BjuA016924	Chlorophyll a-b binding protein 1	Q9C5R6	Photosynthesis
12	*BjuNTF2A*	NA	Nuclear transport factor 2A	Q9FZK4	Nucleocytoplasmic transport
13	*BjuATPs*	NA	ATP synthase subunit gamma	Q0WWB3	Proton-transporting ATP synthase activity
14	*BjuAIP*	BjuA033702	Aluminum-induced protein	Q56ZC9	Uncharacterized protein
15	*BjuWSCP1*	NA	Water-soluble Chlorophyll protein	Q67ZM3	Putative drought induced protein
16	*BjuUBQ*	NA	Polyubiquitin	Q3EAA5	Cellular protein modification process
17	*Bju-α-MAN*	BjuA041717	α-mannosidase	Q8LPJ3	Protein deglycosylation
18	*BjuTIF4A*	BjuA006079	Translational initiation factor 4A-1	B9DHY5	Biosynthetic process
19	*BjuGGDR*	BjuB029758	Geranylgeranyl diphosphate reductase	Q9CA67	Chlorophyll biosynthesis
20	*BjuASP2*	BjuB022229	Aspartyl protease family protein 2	Q94BT8	Biosynthetic process
21	*BjuPGU4*	BjuB032977	Polygalacturonase 4	Q0WM21	Biosynthetic process
22	*BjuPAP2*	BjuB014074	Plastid lipid-associated protein 2,	O49629	Abiotic stress response
23	*BjuPGD1*	BjuA003216	Phosphoglycerate dehydrogenase 1	Q56WY7	Biosynthetic process
24	*BjuRMS3*	BjuA039022	Rhamnose biosynthesis 3	Q56Z49	Biosynthetic process
25	*BjuDGK3*	BjuA025293	Diacylglycerol kinase 3	Q8VZG1	Protein phosphorylation and defence response
26	*BjuTPX2*	NA	Thioredoxin-dependent peroxidase 2	D7KT31	Oxidative stress response
27	*BjuGLN1*	BjuA015586	Glutamine synthase1	Q56WN1	Glutamine biosynthetic process
28	*BjuSAdoMet2*	BjuA011370	S-adenosylmethionine synthetase 2	B9DHQ7	S-adenosylmethionine biosynthetic process
29	*BjuACT*	BjuB008540	Actin-2	C0Z223	Cytoskeleton organization
30	*BjuGLY*	NA	Glyoxalase I family	Q9LV66	Uncharacterized protein

NA: Gene locus ID not available

A more in-depth analysis revealed that the majority of the interacting proteins are involved in the biosynthetic processes, which indicate the important role of BjuA.RGS1 in plant metabolism (Table 2). The metabolic changes in the cell wall play an important role in plant development, particularly during fruit ripening and silique maturation [33,34]. These changes are mediated by various cell wall synthesis, loosening, and degrading enzymes, which control the overall dynamics of fruit and silique maturation and dehiscence. In our study, multiple cell wall synthesis and degrading enzymes like BjuEXP6, Bju-α-MAN, BjuPGU4, and BjuRMS3, were identified as the interacting partner for BjuA.RGS1box+Ct domain. Interaction of these proteins could possibly activate the heterotrimeric G-protein signaling and their associated downstream components, which eventually regulate the cell wall metabolism during the pod maturation stage in *Brassica* species. Earlier, Klopffleisch et al. [17] through Y2H library screening also identified various interacting partners of G-protein components that are involved in cell wall metabolism. Besides, the identification of interacting proteins like BjuGGDR, BjuASP2, BjuGLN1, and BjuSAsoMet2 in the current study suggests the probable involvement of the identified BjuRGS1-targets in other biosynthetic processes of *B. juncea*.

Furthermore, various kinases like BjuMEK4, BjuDGK3, and serine/threonine-protein kinase were also found to interact with the BjuA.RGS1box+Ct domain (Table 2). In plants, phosphorylation of RGS protein by RLKs, WNK, and other kinases are likely to activate the downstream G-protein signaling [14,35–38]. The protein kinases identified in the present study could play an important role in the phosphorylation of BjuA.RGS1, resulting in its separation from cognate Gα protein, thereby reinforcing phosphorylation-based activation and deactivation regulation of the G-protein cycle in plants, which needs further investigation.

**Figure 3:**
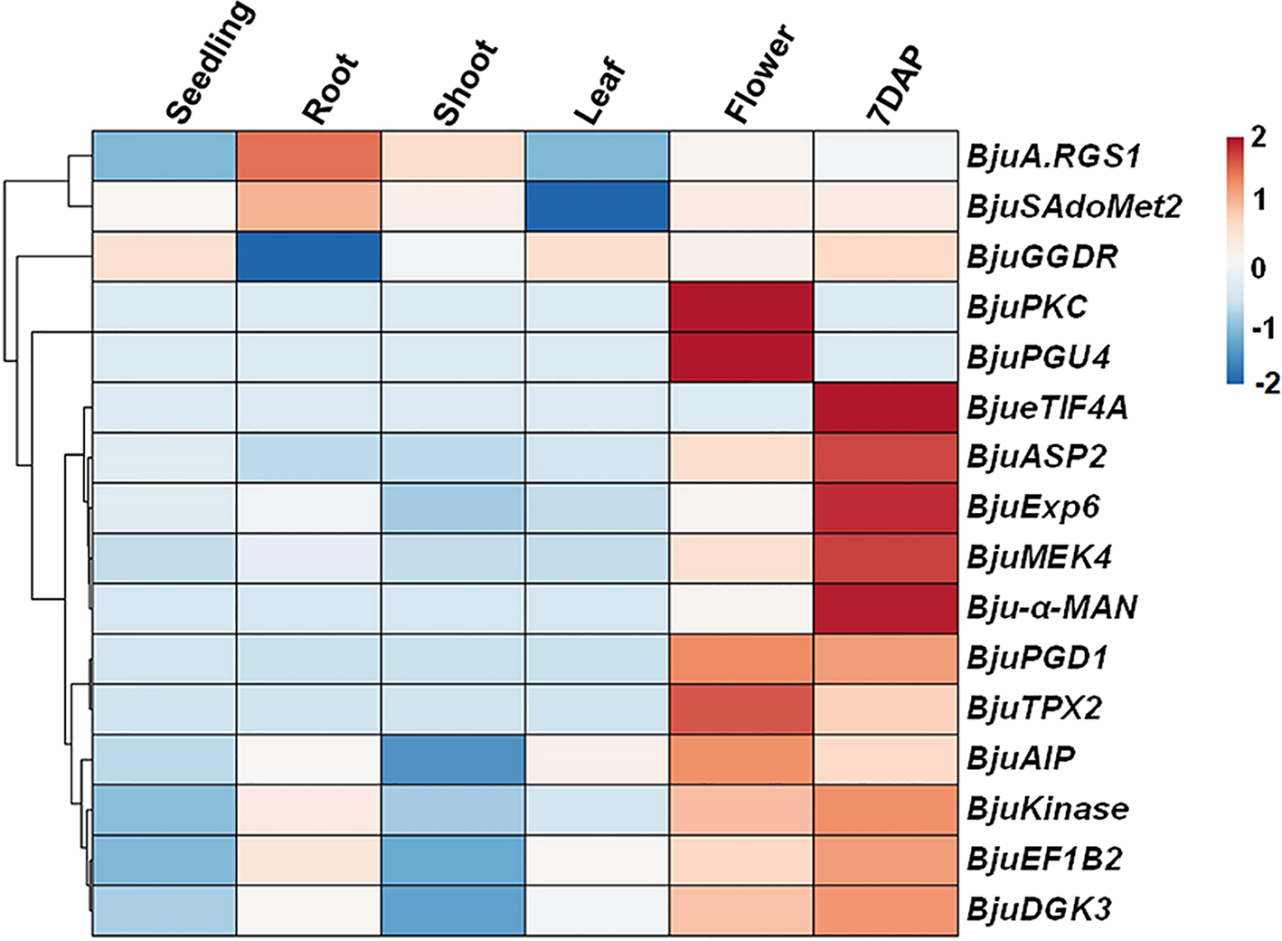
Transcript expression analysis of BjuA.RGS1box+Ct domain-interacting proteins during different developmental stages of *B. juncea* The expression data were normalized against *B. juncea* Actin (Set at 100). Rows are clustered using correlation distance and average linkage. The heat map represents the hierarchical clustering of the log (x) transformed value of BjuA.RGS1box+Ct domain-interacting protein expression. The color scale represents the average signal, which is shown right on the heatmap. Both clustering and heatmap analysis was carried out using the R-based web tool, ClustVis (https://biit.cs.ut.ee/clustvis/).

The proteins that are interacting with each other could possess similar biological functions, and cellular localization, and are more likely to be coexpressed [39]. To investigate the coexpression pattern, we analyzed that the expression of selected BjuA.RGS1-interacting proteins represents various classes of biological functions, in different tissue types of *B. juncea* (Figure 3). The *BjuEF1B2*, *BjuExp6*, *BjuAIP*, *BjuGGDR*, and *BjuSAdoMet2* genes show profound expression in different tissue types. Besides, a few interacting genes like *BjuKinase*, *BjuASP2*, *BjuPGD1*, and *BjuDGK3* were found to be predominantly expressed during flowering and silique stages, suggesting their possible involvement with BjuRGS proteins in plant reproduction.

In summary, we identified multiple RGS-encoding genes in the allotetraploid *B. juncea*, displaying distinct transcriptional differentiation during plant developmental stages. Using the Y2H approach, a total of 30 BjuA.RGS1-interacting proteins were identified that are involved in diverse biological and cellular functions, and primarily associated with biosynthetic and signaling transduction processes. Furthermore, in-depth characterization of these interacting proteins will help in establishing their role in RGS-dependent and independent G-protein signaling processes specific to plant lineage.

## Supplementary Material

Supplementary Figures S1-S2 and Tables S1-S2

Supplementary Table S3

## Data Availability

Data associated with the paper can be accessed by contacting the authors.

## Abbreviations

3-AT: 3-Amino-1.2.4-triazole
7-TM: seven transmembrane
BD: binding domain
BRAD: Brassicaceae Database
CDS: coding DNA sequences
CFU: colony-forming unit
Ct: C-terminal region
DAP: days-after-pollination
DDO: double drop-out
GO: gene ontology
GPCR: G-protein coupled receptor
NCBI: National Center for Biotechnology Information
PlantGSEA: Plant GeneSet Enrichment Analysis
PPI: protein–protein interaction
QDO: quadruple drop out
RGS: Regulator of G-protein Signaling
TAIR: The Arabidopsis Information Resource
Trp: tryptophan
WGT: whole-genome triplication
Y2H: Yeast-two-hybrid
