# Supplementary material for: Interacting partners of Brassica juncea regulator of G-protein signaling protein suggest its role in cell wall metabolism and cellular signaling
Source: Biosci Rep. 2022 Jul 14;42(7):BSR20220302. doi: 10.1042/BSR20220302 (PMC9284343; doi:10.1042/BSR20220302)
Supplement: Supplementary Figures S1-S2 and Tables S1-S2 [file BSR-2022-0302_supp.pdf]

SUPPLEMENTARY DATA

Title: Interacting partners of *Brassica juncea* Regulator of G-protein Signaling protein suggest its role in cell wall metabolism and cellular signaling

Authors: Roshan Kumar and Naveen C. Bisht

The supplementary data section contains 3 tables and 2 figures.

Table S1: Primers used for gene amplification, expression analysis, and interaction studies.

Table S2: Amino acid sequence identity (%) of deduced *B. juncea* RGS proteins with corresponding protein sequence from *Arabidopsis* (AtRGS1).

Table S3: Distribution of BjuA.RGS1box+Ct domain interacting proteins, as separated into biological process (BF_BjuRGS), molecular function (MF_BjuRGS) and cellular component (CC_BjuRGS). [Provided as an excel file]

Fig. S1: Confirmation of interaction of BjuA.RGS1box+Ct domain with its partners.

Fig. S2: A graphical view of the Gene Ontology (GO) distribution of BjuA.RGS1box+Ct domain interacting proteins.

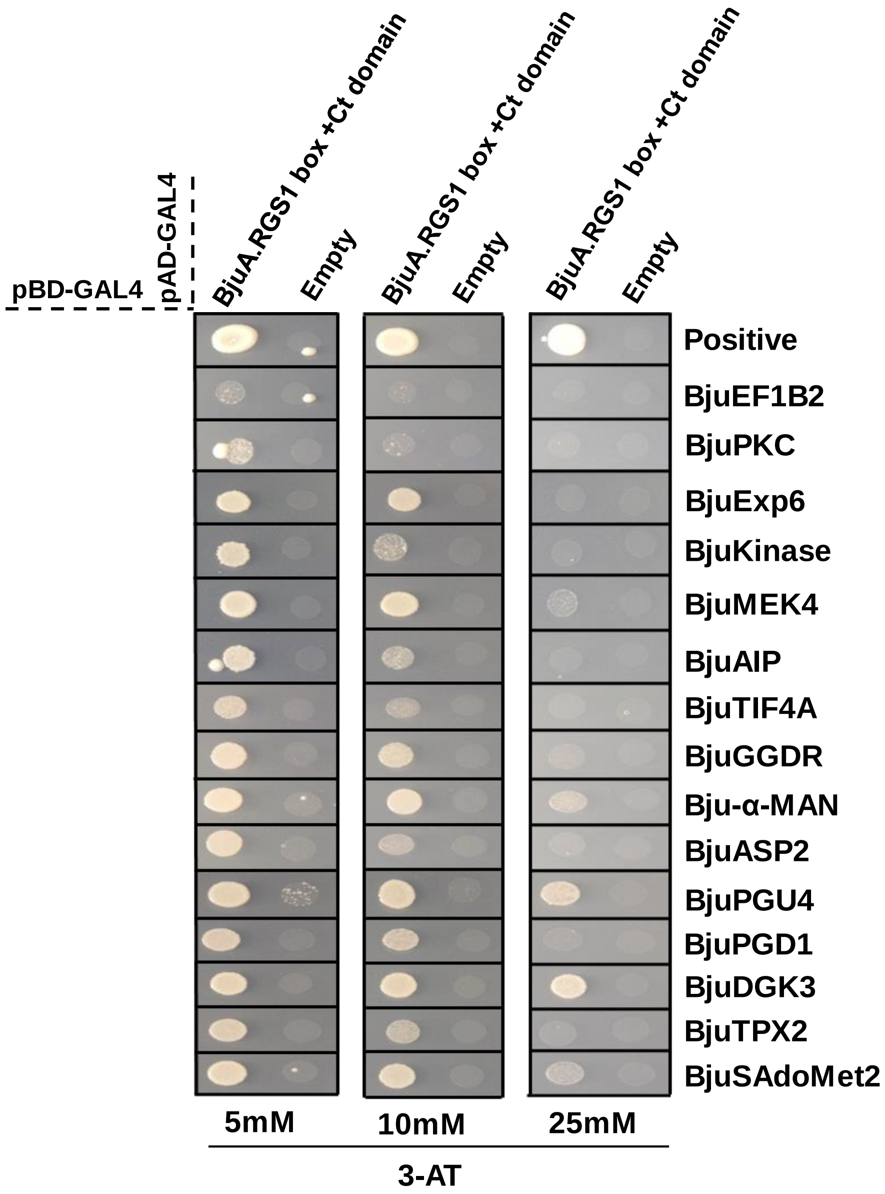
pBD-GAL4
pAD-GAL4
BjuA.RGS1 box +Ct domain
Empty
BjuA.RGS1 box +Ct domain
Empty
BjuA.RGS1 box +Ct domain
Empty
Positive
BjuEF1B2
BjuPKC
BjuExp6
BjuKinase
BjuMEK4
BjuAIP
BjuTIF4A
BjuGGDR
Bju-α-MAN
BjuASP2
BjuPGU4
BjuPGD1
BjuDGK3
BjuTPX2
BjuSAdoMet2
5mM
10mM
25mM
3-AT

Fig. S1: Confirmation of interaction of BjuA.RGS1box+Ct domain with its partners. Bait and prey plasmids were co-transformed into Y2HGold yeast cells. The transformants were placed on the QDO medium but containing different concentrations (5, 10 and 25mM) of the 3-Amino-1.2.4-triazole (3-AT). Co-transformation of pGBKT7-53 and pGADT7-T acted as a positive control, while co-transformation of pGBKT7-empty vector and pGADT7-prey plasmids was used as empty control.

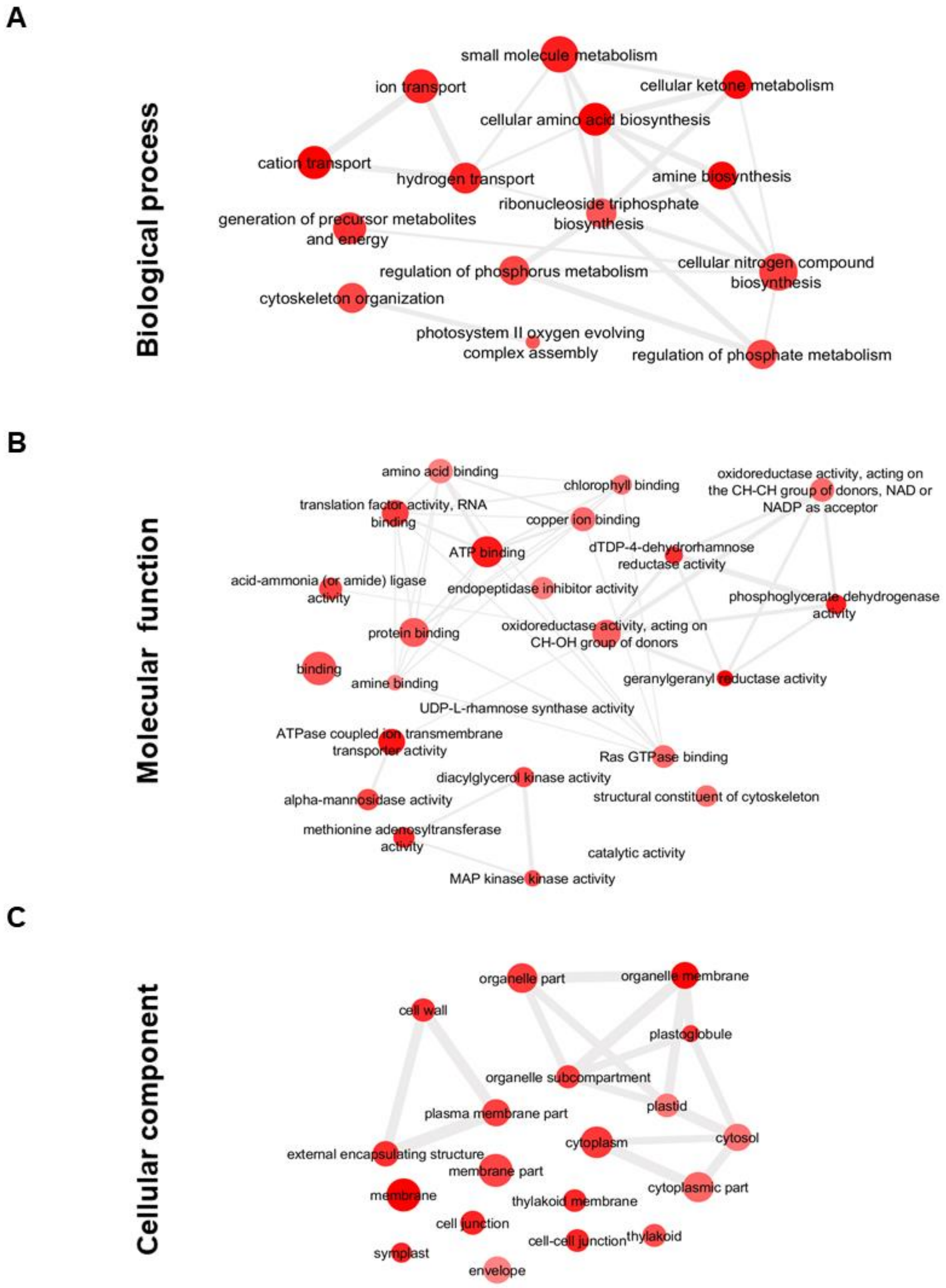
A
Biological process
small molecule metabolism
ion transport
cellular ketone metabolism
cellular amino acid biosynthesis
cation transport
hydrogen transport
amine biosynthesis
ribonucleoside triphosphate biosynthesis
generation of precursor metabolites and energy
regulation of phosphorus metabolism
cellular nitrogen compound biosynthesis
cytoskeleton organization
photosystem II oxygen evolving complex assembly
regulation of phosphate metabolism
B
Molecular function
amino acid binding
chlorophyll binding
oxidoreductase activity, acting on the CH-CH group of donors, NAD or NADP as acceptor
translation factor activity, RNA binding
copper ion binding
ATP binding
dTDP-4-dehydrorhamnose reductase activity
acid-ammonia (or amide) ligase activity
endopeptidase inhibitor activity
phosphoglycerate dehydrogenase activity
protein binding
oxidoreductase activity, acting on CH-OH group of donors
binding
amine binding
geranylgeranyl reductase activity
UDP-L-rhamnose synthase activity
ATPase coupled ion transmembrane transporter activity
Ras GTPase binding
diacylglycerol kinase activity
alpha-mannosidase activity
structural constituent of cytoskeleton
methionine adenosyltransferase activity
catalytic activity
MAP kinase kinase activity
C
Cellular component
organelle part
organelle membrane
cell wall
plastoglobule
organelle subcompartment
plastid
plasma membrane part
cytoplasm
cytosol
external encapsulating structure
membrane part
membrane
thylakoid membrane
cytoplasmic part
cell junction
cell-cell junction
thylakoid
symplast
envelope

Fig. S2: A graphical view of the Gene Ontology (GO) distribution of BjuA.RGS1box+Ct domain interacting proteins. GO were downloaded from Plant GeneSet Enrichment Analysis Toolkit and REVIGO visualized an interactive graph of over-represented GO terms and separated into **(A)** biological process, **(B)** molecular function, and **(C)** cellular component. The detailed GO classification of the BjuA.RGS1box+Ct domain interacting proteins identified in this study is provided in Table S3.

Table S1: Primers used for gene amplification, expression analysis, and interaction studies

Sequence (5' – 3')		Tm ˚C
**Gene amplification primers**		
BjuA/B.RGS1_Dtopo FP	CACCATGGCGAGTGGATGYGCTMWAC	74.4
BjuA/B.RGS1_Dtopo RP	TAACCGGGACTASTGCATCTGGA	68.9
BjuA/B.RGS2_Dtopo FP	CACCATGGCGAGTGGATGTGCTAAACG	77.3
BjuA/B.RGS2_Dtopo RP	TTAACTAGGACTGCTATATCTAGA	52.9
**pGBKT7 cloning primers**		
pGBKT7_ BjuA.RGS1 box +Ct FP	ATTACCATGGCCTCTNCTYTCACARATCAGC	63.8
pGBKT7_ BjuA.RGS1 box +Ct RP	ATTAGAATTCTTAACCGGGACTAGTGCATCT	68.5
**GAL4 BD and AD primers**		
GAL4 DNA BD F.P	TCATCGGAAGAGAGTAGTAAC	55.9
GAL4 DNA BD R.P	CCTCAAGACCCGTTTAGAGG	59.4
GAL4 DNA AD F.P	CTATTCGATGATGAAGATACC	54.0
GAL4 DNA AD R.P	GTGAACTTGCGGGGTTTTTCA	57.9
**Real time primers**		
BjuA.RGS1_RT FP	GATACCTGATAGCGGTTT	50.2
BjuA.RGS1_RT RP	CCTTATCGAATCACCTTCAGGT	63.0
BjuB.RGS1_RT FP	TTCCCAGTTGCAGGCCTTCAC	70.6
BjuB.RGS1_RT RP	GAAGCCAAGAGATATGTA	49.5
BjuA.RGS2_RT FP	GCTTCAAGAACAGACAA	51.3
BjuA.RGS2_RT RP	CGCGAACTCCATAAGCGACTTT	68.2
BjuB.RGS2_RT FP	TGCTTGCCTTCACAAGAGCTG	67.8
BjuB.RGS2_RT RP	CTTGGAAACTCAGAGTGT	52.1
BjuEF1B2 RT FP	GATGCCTGGTCTTCTATG	49.6
BjuEF1B2 RT RP	TACTCGTTGTTAGGTTCAC	49.0
BjuPKC RT FP	AGACTTGGAACTACACAT	47.4
BjuPKC RT RP	GGAAGAAACTCGAAGATG	47.5
BjuEXP6 RT FP	GTGAAAGGAACAAGAACT	46.9
BjuEXP6 RT RP	ATGAAAGTCTGTCCAAAC	47.0
Bjukinase RT FP	ATGCGTTGATGAGTGATT	49.1
Bjukinase RT RP	TGATTCTTCTTCTGCTCTC	48.9
BjuMEK4 RT FP	AAGGTGATCTACGGAAAC	48.8

BjuMEK4 RT RP	GTTCTGGTCGAACATCTC	50.0
BjuAIP RT FP	AAATCTTACAGTCCTTTCC	46.7
BjuAIP RT RP:	TCTTAACCTCAGCATCAA	47.8
Bju-α-MAN RT FP	AATGAAGAGTGTGAAGAG	46.1
Bju-α-MAN RT RP	TATACATGAGAGTGTGAAG	45.6
BjueIF4A RT FP	GTCTTTGGTTATCAACTA	43.3
BjueIF4A RT RP	TTGTAAAACTTCTGGATAT	43.1
BjuGGDR RT FP	AGAATCAGAATCCCAGAT	47.0
BjuGGDR RT RP	ACTTCTTGATGTCACCTT	48.0
BjuASP2 RT FP	ATGAAGACGGTGAATGTT	48.7
BjuASP2 RT RP	CCTTGTTGCTGTATGTTC	48.4
BjuPGU4 RT FP	AAGAGGCGGAGATTCAA	52.0
BjuPGU4RT RP	TACGAAGTTCAATTCCACATC	50.4
BjuPGD1 RT FP	CACAAGACTTCTACGAGCC	52.7
BjuPGD1 RT RP	TTCCTCCGAGAGTCTCAG	52.7
BjuDGK3 RT FP	GCGTGGTTAAGAATAAGAT	47.2
BjuDGK3 RT RP	AAGTCATTGGCTATGTCA	48.1
BjuTPX2 RT FP	ATTTGCTTTAATGTGAATGAT	46.2
BjuTPX2 RT FP	TTGACCTAACACCAAGAC	48.9
BjuAdoMet RT FP	TCTTGTCCAAGTCTCGTA	49.6
BjuAdoMet RT RP	TCAAGTCCAAGTTAATAGTCA	48.5

Table S2: Amino acid sequence identity (%) of deduced *B. juncea* RGS proteins with corresponding protein sequence from *Arabidopsis* (AtRGS1)

	**AtRGS1**	**BjuA.RGS1**	**BjuB.RGS1**	**BjuA.RGS2**	**BjuB.RGS2**
**AtRGS1**	***	88.9	88.9	84.0	85.4
**BjuA.RGS1**		***	95.2	84.6	85.8
**BjuB.RGS1**			***	85.3	86.7
**BjuA.RGS2**				***	91.7
**BjuB.RGS2**					***
